# P-1280. Prevalence of Mupirocin Resistance Among Methicillin-Resistant Staphylococcus Aureus (MRSA) and Methicillin-Susceptible Staphylococcus Aureus (MSSA) from Active Infection. A Surrogate Prevalence for Guiding the Implementation of a Mupirocin Nasal Decolonization Protocol

**DOI:** 10.1093/ofid/ofaf695.1470

**Published:** 2026-01-11

**Authors:** Marly Flowers, Danny Govedarica, Daniel Navas, Jose Alexander

**Affiliations:** AdventHealth, Orlando, FL; AdventHealth, Orlando, FL; AdventHealth Orlando, Orlando, Florida; Advent Health Central Florida, Orlando, FL

## Abstract

**Background:**

The emergence of mupirocin resistance in both Methicillin-Resistant *Staphylococcus aureus* (MRSA) and Methicillin-Sensitive *Staphylococcus aureus* (MSSA) poses a significant challenge for antimicrobial therapy and nasal decolonization. Historically, mupirocin has been effective against *Staphylococcus aureus* strains, but due to the lack of routine susceptibility testing for this agent, its prevalence is not fully understood. However, it has been suggested there is an increasing prevalence of resistance across hospitals. In this prospective study, we are determining the prevalence of mupirocin in MSSA and MRSA isolated from multiple sources of infection.

**Methods:**

913 isolates of *S. aureus* (397=MRSA, 516=MSSA) were isolated from active infections from multiple sources, including respiratory, body fluids, wounds, etc. Tested isolates were collected across a multi-system facility from October 2023 – August 2024, Methicillin resistance was determined by broth microdilution (BMD) for oxacillin and/or cefoxitin screening. Mupirocin screening was performed using a 200µg mupirocin disk diffusion following the disk-based guidelines of the current clinical laboratory standards for high-level of resistance (HLR) and absence of HLR (AHLR).

**Results:**

Out of 913 *S. aureus* isolates, 815 (89.3%) resulted as AHLR for mupirocin (susceptible), and 98 (10.7%) resulted as HLR for mupirocin (resistant). HLR among MRSA was 17.6%, and 5.4% for MSSA. AHLR was observed in 82.4% of MRSA and 94.6% of MSSA

**Conclusion:**

The total prevalence for HLR to mupirocin in circulating infective strains from inpatient and outpatient was 7%. The distribution of HLR between MRSA and MSSA was statistically similar, with 43% and 57%, respectively. This distribution indicates there is no correlation between methicillin resistance and mupirocin resistance; that was expected since both resistance mechanisms and agents are different. With a low prevalence of HLR, mupirocin is a viable option for decolonization protocols in clinical settings.

**Disclosures:**

All Authors: No reported disclosures

